# Supplementary material for: Patterns of Intron Gain and Loss in Fungi
Source: PLoS Biol. 2004 Nov 30;2(12):e422. doi: 10.1371/journal.pbio.0020422 (PMC532390; doi:10.1371/journal.pbio.0020422)
Supplement: Table S1 — Also available at http://genes.mit.edu/NielsenEtAl/. (4.3 MB ZIP). [file pbio.0020422.st001.zip › NielsenEtAl/html/334.html]

AN0240.1.NCU02136.1.MG02624.1.FG08723.1


```
 CLUSTAL W (1.82) Multiple Sequence Alignments - Introns Inserted


Sequence 1: MG02624.1	324 aa
Sequence 2: FG08723.1	323 aa
Sequence 3: AN0240.1	324 aa
Sequence 4: NCU02136.1	324 aa
Alignment Length: 324 aa
Number Identitical Residues: 228 aa
Alignment Score (without introns) 9443


MG02624.1 	MSNSLDQLKSSGT~TVVCDSG1DFAT~IGKYKPQDATTNPSLILAASKKAEYAKLIDDAI
NCU02136.1	MSNSLEQLKATGT0VVVCDSG1DFAT~MAKYKPQDATTNPSLILAASKKPEYGALLDAAI
FG08723.1 	MSSALDQLKATGT0TVVSDSG1DFAS1IGKYKPQDATTNPSLILAASKKEEYAKLIDVAI
AN0240.1  	MSSSLEQLKATGT0VVVCDSG~DFAT~IDKYKPQDATTNPSLILAASKKPEYASLIDAAV
          	**.:*:***::** .**.*** ***: : ******************** **. *:* *:

MG02624.1 	AYAKKQGGSVDDQVDASLDRLLVEFGKEILKIIPGKVSTEVDARFSFDTKASVDKALHII
NCU02136.1	ADAKKGPGSPEEKVDAALDYLLVQFGKKILEIVPGKVSTEVDAALSFDTKASVDKALHII
FG08723.1 	DYAKQKGGSVDQQVDDALDRLLVEFGKEILKIIPGKVSTEVDARYSFDTEASVNKALHLI
AN0240.1  	EKGKKQGGSVDDQVDATLDYLLVEFGKKILDIIPGKVSTEVDARFSFDTKASVDKALHII
          	  .*:  ** :::** :** ***:***:**.*:**********  ****:***:****:*

MG02624.1 	K~LYEAEGISKDRVLIKIASTWEGIKAAEILQRDHGINCNLTLMFSLVQAIAAAEADAFL
NCU02136.1	K0LYEAEGISKDRILIKIASTWEGIKAAEILQRDHGINCNLTLMFSLTQAIAAAEAGAFL
FG08723.1 	E0LYGEQGISKDRILIKIAATWEGIKAAEILQRDHGINTNLTLMFSLVQAIGAAEAGAYL
AN0240.1  	K0LYEEAGISKDRILIKIASTWEGIQAAHILQSQHGINCNLTLMFSLVQAIAAAEAGAFL
          	: **   ******:*****:*****:**.*** :**** ********.***.****.*:*

MG02624.1 	ISPFVGRILDWYKAAHKKEYKKEEDPGVESVKNIFNYYKKFGYKTIVMGASFRNTGEITE
NCU02136.1	ISPFVGRILDWFKAHTGKNYSKEEDPGVASVKTIFNYYKKFGYKTIVMGASFRNIGEITE
FG08723.1 	ISPFVGRILDWFKASTKKEYTKEEDPGVLSVKQIFNYYKKFGYNTIVMGASFRNTGEITE
AN0240.1  	ISPFVGRILDWYKAAHKRDFSKEEDPGVKSVQSIFNYYKKFGYKTIVMGASFRNVGEITE
          	***********:**   :::.******* **: **********:********** *****

MG02624.1 	LAGCDYLTIS0PNLLEELLNSSEAVPKKLDAQGAAALDIQKKTYISDEPLFRFDFNEDQM
NCU02136.1	LAGCDYLTIS0PNLLEELLNSDAPVPQKLNAADAASLSIEKKTYINSEPTFRFDFNEDQM
FG08723.1 	LAGCDYLTIS0PNLLEDLLNSSDSVPKKLDASQASSLDIEKKSYIKDEALFRFDFNEDQM
AN0240.1  	LAGCDYLTIS0PGLLEELYNSKDAVPKKLDAASATTLDITKRSYLNDEALFRFDFNEEAM
          	********** *.***:* **. .**:**:*  *::*.* *::*:..*. *******: *

MG02624.1 	AVEKLREGISKFAADADTLKGILKEKIQA
NCU02136.1	AVEKLREGISKFAADAVTLKEIIKQKLAE
FG08723.1 	AVEKLREGISKFAADAVTLKGILKEKLS-
AN0240.1  	AVEKLREGISKFAADAVTLKDILKQKVQA
          	**************** *** *:*:*:
```
